# Do Preventive Programs Reduce the Need for New Sedations for the Treatment of Oral Pathologies in Healthy and Special Health Care Needs Children?

**DOI:** 10.3390/jcm13185366

**Published:** 2024-09-10

**Authors:** Inmaculada Gómez-Ríos, Clara Serna-Muñoz, Amparo Pérez-Silva, Yolanda Martínez-Beneyto, Gabriele Di Carlo, Antonio José Ortiz-Ruiz

**Affiliations:** 1 Department of Integrated Pediatric Dentistry, Faculty of Medicine, University of Murcia, 30008 Murcia, Spain; macu@innovadental.com (I.G.-R.); claraserna@live.com (C.S.-M.); yolandam@um.es (Y.M.-B.); ajortiz@um.es (A.J.O.-R.); 2 Department of Oral and Maxillo-Facial Sciences, University of Rome Sapienza, 00185 Rome, Italy; gabriele.dicarlo@uniroma1.it

**Keywords:** pediatric dentistry, sedation, prevention program, oral health, special health care needs

## Abstract

**Background:** The goal is to analyze the need for reinterventions under deep sedation to treat oral pathologies in a population of children with special health care needs (SHCNs) and healthy children who followed a prevention program and to study the influence of parental motivation and child collaboration on the need for reinterventions under deep sedation. **Methods:** A retrospective study was carried out in a private clinic in Cartagena (Murcia, Spain), with patients treated under deep sedation from 2006 to 2018, both years included, following the Strobe statement. **Results:** In this study with 230 children who were treated under deep sedation, 23.92% underwent two or more sedations. The mean time elapsed between the first and the second sedations was 21.64 ± 15.87 months, and the main cause for reinterventions was the occurrence of new pathologies. Significantly more pulp treatments were performed in the first sedation than in the second (*p* = 0.013) and in the third (*p* = 0.007). Healthy children required fewer reinterventions under deep sedation than children with special needs (6.42% vs. 39.67%). Similarly, patients who followed the preventive program and required some type of dental treatment were reoperated fewer times than those who did not follow the preventive program (35.8 vs. 50%); than “cooperative” children as opposed to “non-cooperative” (12.12% vs. 60.93%) and than patients with “motivated” parents as opposed to those with “non-motivated” parents (20.83% vs. 46.34%). A total of 50% of the children who participated in the preventive program became “cooperative”, and 100% were able to receive some treatment in the dental chair. **Conclusions:** Prevention programs, including motivational interviewing, are essential to improve children’s behavior in the dental chair and reduce the need for reinterventions under general anesthesia or deep sedation. Although patients with special needs do require more sedation during their lifetime due to their inability to cooperate, these programs are necessary for the maintenance of oral health status and for the early diagnosis of caries lesions. Pediatric dentists should implement a quarterly preventive program because it improves patient collaboration. It is essential to achieve the motivation of parents in the oral care of their children.

## 1. Introduction

The World Health Organization (WHO) includes caries as one of the main health disorders worldwide and proposes a reform of health systems in oral health, aimed at paying more attention to prevention and minor treatments and avoiding invasive dental treatments [1]. The policies and guidelines of the main international pediatric dentistry associations (AAPD, EAPD and IAPD) state the need to encourage oral health professionals and caregivers to implement preventive practices that can stop caries early and improve individual and public dental health [2,3,4]. Thus, it is imperative that children have their first contact with a dentist, at the latest, at 12 months [5] and begin to evaluate their caries risk assessment. Caries risk assessment is the determination of the likelihood of an increase in caries incidence over a given period or the likelihood of a change in the size or activity of the lesion already present [6]. Since repairing caries lesions does not stop the disease and restorations have a limited lifespan, prevention along with minimally invasive treatments and individualized treatment plans based on caries risk are the three basic pillars of the new approach to the disease. This new approach to caries treatment is the basis for various protocols developed by scientific associations [2,3,4,5,6,7].

Children with special health care needs (SHCNs) should be treated in the same way and referred to the appropriate centers if treatment under general anesthesia is required [8]. Prevention and follow-up programs are also needed to motivate parents and caregivers to better control diet and hygiene. Creating a close relationship among the patient, parents, caregivers and their pediatric dentist (“dental home”) will help to implement individualized preventive measures [9].

The treatment of dental pathologies on a single day is justified, as it is associated with a clear increase in the patient’s quality of life [10]. Despite this, reoperations under sedation or general anesthesia are not uncommon. Among the possible causes of the need for retreatment under surgical approach, some authors point to the initial oral health status of the sample and the prevention plan [11] and others to the general health status, the treatment received in the first intervention, fewer scheduled prevention appointments or the need for more emergency appointments [12]. A recent review of the literature [13] found that few studies have evaluated preventive programs implemented after treatment under general anesthesia and their effect on the need for further interventions.

The aims of our study were as follows:

1. To analyze the need for reinterventions with deep sedation to treat oral pathologies in a population of healthy children and SHCNs who followed a preventive program consisting of a three-month check-up with plaque control, fluoride application, diet control, recall of hygiene techniques, motivational interviewing and control of fear of the dentist.
2. To study the influence of parental motivation and child collaboration on the need for reinterventions under deep sedation.

Our null hypothesis is that for patients treated under deep sedation or general anesthesia, participating in a caries prevention plan does not influence the need for new oral intervention under sedation.

## 2. Materials and Methods

This article was written according to the STROBE statement (www.strobe-statement.org) (accessed on 25 October 2023). A retrospective study was conducted in a private clinic in Cartagena (Murcia, Spain), analyzing the evolution of patients treated under deep sedation during the years 2006 to 2018, both years included. Inclusion criteria were age 2–18 years; optimal general health status, which we will refer to as “healthy children”, or children with special needs (SHCNs) [14]. Of the 274 potential patients, the final sample is 230 children after discarding medical records that were not correctly filled in. Parents/guardians of all patients had signed an informed consent and received an information sheet. The study was approved by the Research Ethics Committee of the University of Murcia (ID:2034/2018).

The anesthetic procedures to treat oral pathologies were performed by a team of anesthesiologists and nurses. Dental interventions and patient follow-ups were performed by the same dentist and dental hygienist, regardless of the type of patient. In the first visit, as well as in the subsequent appointments, a motivational interview was carried out where we worked with the parents on hygiene habits at home (hygiene techniques, recommendation of the adequate amount of toothpaste according to age and use of toothpaste with fluoride concentrations above 1450 ppm) and on diet (gradual changes in the child’s diet after a diet study), and we insisted on the need for a rigorous follow-up of the patient in the consultation room (according to the caries risk).

The preventive caries check-up was carried out 15–30 days after the intervention under deep sedation. The patients who remained in the clinic were offered to join a preventive program. The preventive program consisted of a periodic check-up as follows: As all patients belonged to the group of children at “high risk” of caries according to the caries management by risk assessment (CAMBRA) [15], the check-up interval was three months with plaque control, fluoride application, diet control, recall of hygiene techniques, motivational interviewing and control of fear of the dentist. Patients who did not comply with the periodicity of the check-ups were not included in this group. The referred patients who returned to their consultations were given a report with the treatments carried out and the recommendation to follow this same protocol in their clinics of origin. The information extracted from the medical records was as follows:

(I) From the first visit
  (A) Demographic data: Age and sex, health status, differentiating between healthy children and children with special needs.
  (B) Reason for sedation.
  (C) Assessment of oral health status prior to the intervention included the following:
    - Hygiene habits. The child was considered to be adherent to oral hygiene instructions when he/she brushed regularly at least twice a day;
    - Presence of plaque on visual inspection on two or more teeth (Yes or No);
    - Presence of tartar on visual inspection on at least one tooth (Yes or No);
    - Presence of caries lesions and number of teeth affected. We considered caries lesions as the loss of enamel integrity (ICDAS 3, 4 and 5);
    - Pulp involvement and number of teeth affected. Pulp involvement was considered to be the presence of ICDAS 6 lesions, nocturnal pain, radiolucent image in radiographs, phlegmons or abscesses;
    - Existence of root debris on visual inspection and number;
    - Absence of teeth due to a dental pathology on visual inspection (number).
(II) On the day of the first operation
  (A) Types of treatments carried out were as follows:
    - Filling;
    - Direct pulp protection;
    - Pulpotomy;
    - Pulpectomy;
    - Endodontics;
    - Apicoforming;
    - Tartrectomy;
    - Scaling and root planing (RAR);
    - Fluoride application;
    - Exodontia.
  (B) Number of teeth treated.
(III) Follow-up
  (A) Attendance at the post-sedation check-up (Yes or No);
  (B) Presence of plaque on visual inspection (Yes or No);
  (C) Need for medication for an oral pathology (Yes or No);
  (D) Improvement at mealtime (Yes or No);
  (E) Attendance in preventive program (Yes or No);
  (F) Cooperative behavior at appointments (Yes or No). Depending on if the patient allowed the dentist and/or hygienist to perform their work in dental chair;
  (G) Motivation of parents in the oral care of their children (Yes or No). Depending on whether or not they are involved in the care of their children’s mouth and implement at home the dietary and oral hygiene recommendations given at the consultation.
  (H) Type of treatments carried out afterward without sedation included the following:
    - Health education;
    - Tartrectomy;
    - Fluoride application;
    - Sealant;
    - Filling;
    - Pulpectomy;
    - Pulpotomy;
    - Preformed crowns;
    - Endodontics;
    - Apicoforming;
    - Exodontia;
    - Space maintainer.
  (I) Year of last revision.
  (J) Follow-up time.
(IV) Reinterventions:
  (A) Reason for reoperation.
  (B) Type of unsuccessful treatment.
  (C) Type of treatment performed included the following:
    - Filling;
    - Sealant;
    - Pulpotomy;
    - Pulpectomy;
    - Endodontics;
    - Apicoforming;
    - Tartrectomy and fluoride application;
    - Preformed crowns;
    - Space maintainer;
    - Exodontics.
  (D) Number of sedations.
  (E) Time from first to last sedation.

### Statistical Analysis

All data were collected in an Excel sheet and were statistically analyzed with R version 3.6.0. (R Core Team 2019) by the Scientific and Technical Research Area, Statistical Support Section (Edificio SACE, ground floor 30100. Espinardo Campus. University of Murcia). A descriptive analysis of all study variables was performed. Continuous quantitative variables were compared by using *t*-tests, *t*-test with Welch’s correction or Mann–Whitney test according to the assumptions of normality and homoscedasticity. To establish the relationship between discrete qualitative or quantitative variables, contingency tables were performed with Pearson’s or Fisher’s exact tests, depending on if the corresponding assumptions were met. To determine the equality of proportions, a “test of equality of proportions without continuity correction” was used. A *p* < 0.05 was considered significant.

## 3. Results

Out of the 230 patients operated on, 145 (63.05%) were referred from other dental clinics for treatment under deep sedation. In the total sample (*n* = 230), 61.74% were male and 38.26% were female. The mean age was 7.10 ± 3.40 years [25th and 75th quartiles of 4 and 9 years]. The ages with the highest number of children were 4 (*n* = 31), 6 (*n* = 29), 7 (*n* = 25), 8 (*n* = 25) and 9 (*n* = 25) years. A total of 47.40% were healthy patients (*n* = 109), and 52.60% (*n* = 121) had special needs. In the group of healthy children, the mean age was 5.04 ± 2.42, and in the group of children with special needs, it was 8.95 ± 3.09 (Table 1).

The main reason for treating the patient under sedation was poor handling in the dental chair (99.5%; 229/230). A total of 79.57% of the sample had no dental hygiene habits at home; 90.86% had dental plaque, and 90.87% had lesions of dental caries. A total of 45.22% of the patients had 5 to 10 teeth affected by caries lesions. Pulp pathology was diagnosed in 67.83% of the patients, with one or two teeth affected in 40.86% of the children. A total of 4.34% had missing teeth, and 13.91% had root remains. There was a significantly higher percentage of pulp involvement, a higher number of teeth with caries lesions per child, and a higher number of teeth with affected pulp per child in the group of healthy children and a higher percentage of children with tartar in the group of children with special needs (Table 2).

Of the 230 children treated under deep sedation, 175 patients (76.08%) underwent a single sedation and 23.92% (*n* = 55) underwent two or more sedations. The number of children who underwent successive sedation and the mean time elapsed between the first and the remaining sedations are presented in Table 3.

**Figure 1:**
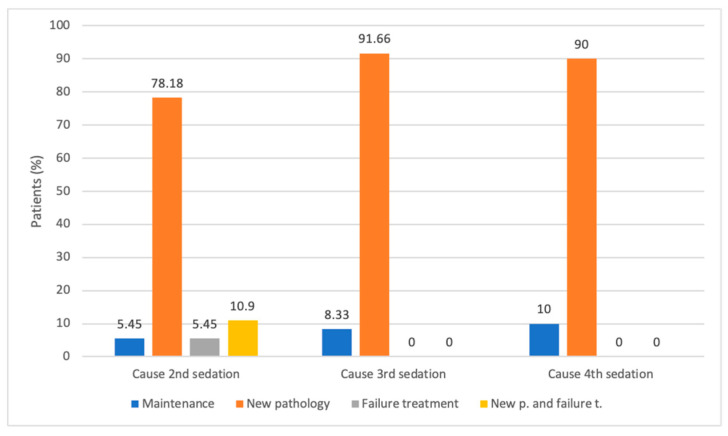
Cause of the consecutive sedations. (New p.: new pathology. Failure t.: failure of treatment).

The main cause for reinterventions was the occurrence of a new pathology. Only three patients were sedated a second time due to failure of previous treatments (Figure 1).

Considering the total sample, significantly more pulp treatments were performed in the first sedation than in the second (*p* = 0.013) and in the third (*p* = 0.007). Healthy children required fewer reinterventions under deep sedation than children with special needs (6.42% vs. 39.67%, two sedations; 2.75% vs. 17.35%, three sedations; 0.008% vs. 7.44%, four sedations). If we analyze the treatments performed according to the patient’s state of health, we observe that during the second sedation, patients with special needs underwent more obturations and endodontics and healthy patients underwent more pulpectomies and extractions. And during the third sedation, patients with special needs underwent more pulpectomies, sealants and tartrectomies and/or fluoride applications and healthy patients underwent more obturations and extractions. There are no pulp treatments in either group during the fourth sedation (Table 4).

**Figure 2:**
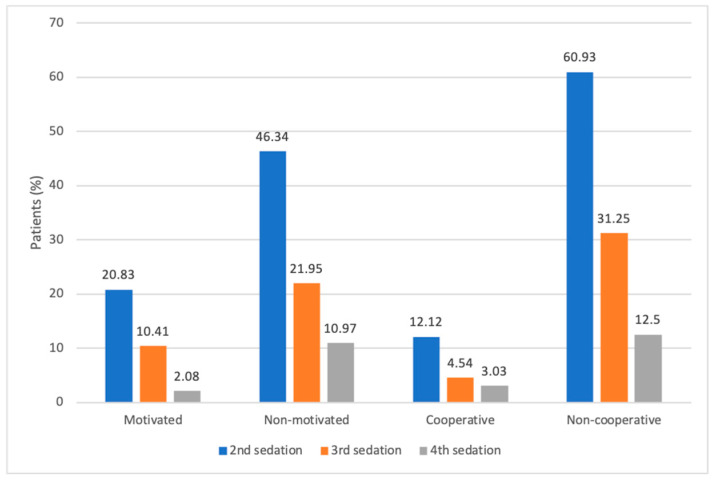
Percentage of patients reintervened under deep sedation based on whether parents are motivated or not about oral care of the children and whether the child is cooperative or non-cooperative.

A total of 81 of the patients were referred by other dentists, and 85 of the clinic’s own patients decided to follow up with us (*n* = 166). Let us remark that we cannot track or follow up the patients that go back to their referrer, so the remaining (*n* = 166) make the sample where the effect to participate or not in a prevention program in the need for reinterventions could be studied. In 130 of them, we recorded if they were “cooperative” (patient who allowed the dentist and/or hygienist to perform their work in dental chair) (*n* = 66) or “non-cooperative” (*n* = 64) and whether their parents were “motivated” (are involved in the care of their children’s mouth and implement at home the dietary and oral hygiene recommendations given at the consultation) (*n* = 48) or “non-motivated” (*n* = 82) with the care of their children’s mouths. Patients with “motivated” parents underwent fewer second sedations than children with “non-motivated” parents (20.83% vs. 46.34%; *p* = 0.006) and fewer third (10.41% vs. 21.95%; *p* = 0.15) and fourth (2.08% vs. 10.97%; *p* = 0.089) interventions (Figure 2).

“Non-cooperative” children needed to be sedated a second time more often than “cooperative” children (60.93% vs. 12.12%; *p* < 0.001). The same was true for third sedation (31.25% vs. 4.54%; *p* = 9.9 × 10^−5^) and fourth sedations (12.50% vs. 3.03%; *p* = 0.061). In addition, “non-cooperative” patients or those with “non-motivated” parents needed to be sedated for simple treatments (tartrectomy, fluoride application and/or sealants), while “cooperative” children or those with “motivated” parents were able to receive these treatments in the dental chair.

**Figure 3:**
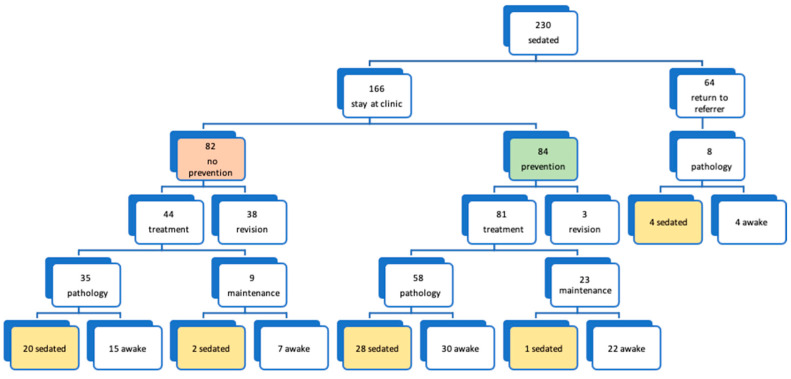
Evolution of the patients after the first sedation depending on whether or not to continue in the dental clinic and whether or not to carry out the prevention follow-up program. Percentages are computed only for patients that do not return to referrer.

The 166 patients following up in the clinic after the first intervention were advised to follow a quarterly preventive program, with an average time of 39.6 months. This was performed by 52.23% of the healthy patients and 49.99% of the patients with special needs. Of the 84 patients who followed the preventive program and required some type of dental treatment, only 35.8% needed to undergo treatment again under deep sedation compared to 50% of the children who did not follow the preventive program (Figure 3).

A total of 53.57% of the children who participated in the preventive program became “cooperative”; 100% were able to receive some treatment while awake in the dental chair, compared to 43.90% of the patients who did not follow the prevention program (*p* < 0.001) which included the following: dental surgery (44.04% vs. 25.61%; *p* = 0.0127), maintenance (85.71% vs. 28.04%; *p* < 0.001) and health education (97.61% vs. 35.36%; *p* < 0.001). A total of 80.00% of healthy patients who followed the preventive program were able to receive awake operative treatments, compared to 40.60% of healthy children who did not follow the program. However, patients with SHCNs who followed the preventive program were not able to receive awake operative treatments, although they were able to receive maintenance treatments (Table 5).

## 4. Discussion

The treatment of all dental pathologies under general anesthesia or deep sedation on a single day is justified because it improves patients’ quality of life [10]. However, these patients often require reinterventions for the treatment of new pathologies or failures of previous treatments [11,12,17]. Of the 230 healthy SHCNs patients in our study, 76.08% were sedated only once, but the remaining 23.92% (55 patients) required a second intervention under deep sedation within a mean time of 21 months. This percentage was higher than that of the study by Tahmassebi, Achol and Fayle [18] which had a percentage of second sedations of 12.9% at 13–24 months after the first sedation, probably because SHCNs patients had more extractions than fillings in the first intervention; thus, decreasing the number of teeth in the mouth also decreased the possibility of new disease. König et al. [17], with a higher mean number of extracted teeth per child (3.7 vs. 0.64 in healthy children and 1.35 in SHCNs [17]) and a higher percentage of healthy patients than our study (71.0% vs. 47.4% [16]), recorded a reintervention rate of 11%. Other studies, such as Rudie et al. [19] and Guidry et al. [12], recorded fewer second sedations (9.0% and 4.9%, respectively) possibly due to the lack of a standardized follow-up protocols. They also observed that children who received more conservative restorative treatment during the first intervention tended to need more retreatments under general anesthesia [20]. However, in our study, neither the initial oral pathology nor the treatments performed at the first deep sedation were significantly related to the need for reoperation under sedation.

Considering the health status of the child, in our study, 6.42% of healthy patients required a second sedation. Only the study by Kwok-Tung et al. [21], where exodontia accounted for 41% of the total number of treatments performed in the first intervention, had a lower percentage of healthy children who underwent a second intervention (3.5%). However, other authors [22,23] describe higher values, ranging from 11% to 18.8%, due to treatment failures (performing many pulp treatments without using a rubber dam [22]), non-attendance of patients at check-up appointments or persistent non-cooperation of the child in the chair. A second sedation was required in 39.67% of the SHCNs in our study. This percentage is higher than in the studies reviewed, whose sample consists only of children with SHCNs [11]. Bücher et al. [11] explained their low percentage (10.8%) of reinterventions because the initial sample had a very low dmft (decayed, missing, and filled primary teeth), because they rarely performed pulp treatment in the deciduous dentition and because the mean number of extractions in the first intervention was 2.5 teeth per patient, compared to 1.35 in our study [16]. In our opinion, the reason for the high percentage of second sedations in our study was the quarterly prevention program, which allowed very close monitoring of the patients who voluntarily chose to join it. Motivational interviews were carried out at these appointments, and different preventive aspects such as hygiene techniques or how to make changes to the child’s diet were discussed with the parents. In the case of SHCNs patients, increased follow-up did not necessarily mean less pathology, as preventive measures in the clinic are not effective if they are not accompanied by good hygiene and daily diet control [11]. However, carrying out check-ups did allow early detection of new oral pathologies and the need for its treatment, and we were able to incorporate interventions under deep sedation as another tool for the correct maintenance of oral health, also considering that our regional public health system assumes all treatments performed in the operating theatre for children with SHCNs, while treatments of the deciduous dentition performed without general anesthesia entail a cost for parents. Parents of children with disabilities who have received more than one treatment under general anesthesia tend to repeat this approach [24].

One of the main goals of the preventive program is to avoid further interventions under general anesthesia [12,17,25,26] by managing the child’s behavior in the dental chair [27,28]. Few studies have analyzed the long-term effect of such programs on the occurrence of new pathologies and on the need for subsequent reoperations under general anesthesia or deep sedation, and their results are contradictory. Thus, Almeida et al. [20] who compared the evolution of healthy children with ECC (Early Childhood Caries) treated under sedation with another group of children who did not present caries, performing check-ups every 6–9 months for 2 years, observed that only 38% of the group of children with ECC attended all check-ups, that children with a history of ECC had more recurrent caries than healthy children and that there was no relationship between the frequency of check-ups and the need for second sedations (17%) or with the appearance of new lesions. In contrast, other authors [12] found that missed check-ups increased the occurrence of new caries lesions, while attendance at check-ups decreased the risk of a second operation in the operating theatre. Raja et al. [29], in a sample of children aged 2–5 years, treated under general anesthesia for extractions, observed a high incidence of caries lesions in the first permanent molars and a low number of sealants two years after treatment under general anesthesia, due to the lack of attendance of patients at their referral clinics for preventive treatment, with only 14.39% of patients attending check-ups every 6 months.

The patients in our study who attended the preventive program were sedated for the second time more than those who did not undergo prevention (34.5% vs. 27%) due to the fact that during check-ups, we diagnose more pathologies and need for treatment. Despite our efforts to teach hygiene techniques and healthy dietary habits, this was not enough [28] and our patients continued to develop new lesions [20]. Olley et al. [30] showed that lack of brushing at home is one of the causes of failure [11] and although 78% of parents were interested in the preventive program, they stated that they did not have the time or energy to fight with their children and felt social pressure to consume sugary food. A high periodicity of screening is very important to achieve behavioral change in a household [31].

Even though our quarterly preventive program for children at high risk of caries did not lead to the complete disappearance of the disease, it was useful to teach the child to be cooperative in the dental chair which is one of the necessary requirements to avoid further sedation [28]. Of the total number of patients who followed the preventive program, 53.5% became compliant. In the case of healthy patients, 80% were able to perform operative treatments awake. In patients with special needs, the preventive follow-up was more oriented toward the maintenance of oral health status and early diagnosis of caries lesions than toward avoiding future interventions in the operating theatre [12]. A total of 85% of SHCNs who attended preventive visits can perform maintenance treatments awake, compared to only 32% of those who did not attend regular visits, but when it comes to performing more complex treatments, the percentages are almost equal (18% vs. 16%). Despite this, carrying out the preventive program is justified since these patients need to be treated under deep sedation to receive more complicated treatments, being able to perform simpler treatments in the dental chair. Thus, of the patients who attended preventive appointments and required a second sedation, 13.79% underwent pulpectomies compared to 9.09% of those who did not attend preventive appointments and, on the other hand, required fewer fillings (75.86% vs. 90.9%) and tartrectomies and/or fluoride applications (72.41% vs. 95.45%). The main limitation of our study was that it was a retrospective observational study with data from a private clinic where epidemiological research indices such as CAOD and plaque indices are not routinely used, which would have facilitated comparison of the data with other published studies. Another limitation was the lack of information on chronic systemic medications that the children participating in the study may have been taking and that could be contributing factors in the appearance of primary or recurrent caries lesions. However, the parents and caregivers of the children were informed and individualized preventive measures were established to avoid them. A prospective study with exhaustive control of all variables would be ideal.

## 5. Conclusions

Prevention programs that includes motivational interviewing are key to improve children’s dental chair behavior and reduce the need for reinterventions under general anesthesia or deep sedation. Although patients with special needs require more sedations throughout their lives due to their inability to cooperate, these programs are necessary for the maintenance of oral health and for the early diagnosis of new caries lesions.

## Figures and Tables

**Table 1:** Summary of patient demographics and characteristics.

Total Sample Size	230 (100%)
Male	142 (61.74%)
Female	88 (38.26%)
Healthy	109 (47.39%)
SHCNs	121 (52.60%)
Referred patients	145 (63.05%)
Mean age of the total sample	7.10 ± 3.40 (median 7 years)
Healthy	5.04 ± 2.42 years
SHCNs	8.95 ± 3.09 years

**Table 2:** Initial oral health status of healthy children versus children with special needs.

Variable	Total	Healthy	SHCNs	*p*-Value
	(230)	(109)	(121)	
Tooth-brushing habits	20.43%	17.43%	23.14%	0.36 ^b^
	(47)	(19)	(28)	
Plaque	90.86%	93.57%	89.25%	0.35 ^b^
	(209)	(102)	(108)	
Tartar	31.30%	7.33%	52.89%	<0.001 ^b^
	(72)	(8)	(64)	
Carious lesions	90.87%	93.57%	88.43%	0.26 ^b^
	(209)	(102)	(107)	
Pulp involvement	67.83%	78.90%	57.85%	0.0011 ^b^
	(156)	(86)	(70)	
Root remains	13.91%	10.09%	17.36%	0.16 ^b^
	(32)	(11)	(21)	
Missing teeth	4.34%	4.58%	4.13%	1 ^a^
	(10)	(5)	(5)	
Number of teeth with caries lesions per child (mean ± SD)	6.78 ± 4.65	7.49 ± 4.68	6.13 ± 4.54	<0.05 ^c^
Number of teeth with pulpal involvement per child (mean ± SD)	1.84 ± 2.04	2.25 ± 2.01	1.47 ± 2.00	0.0037 ^c^

SHCNs: special health care needs children. *p*-value healthy vs. SHCNs. ^a^ Fisher’s exact test. ^b^ Pearson’s χ^2^ test. ^c^ Mann–Whitney U test.

**Table 3:** Distribution of sedations and corresponding time intervals.

Number of Sedations	Percentage (*n*/N)	Time Media ± SD [Median] (Months)
One	76.08% (175/230)	—
Two	13.47% (31/230)	Second: 21.64 ± 15.87 [21.00]
Three	6.08% (14/230)	Third: 49.43 ± 22.62 [41.50]
Four	2.17% (5/230)	Fourth: 48.00 ± 8.06 [51.00]
Five	1.73% (4/230)	Fifth: 55.50 ± 7.59 [57.50]
Six	0.43% (1/230)	Sixth: 74.00 ± 0.00 [74.00]

**Table 4:** Distribution of treatments among patients with SHCNs and healthy patients based on number of sedations.

Treatments	Total (*n*)				SHCNs (*n*)				Healthy (*n*)			
	1 (230)	2 (55)	3 (24)	4 (10)	1 (121)	2 (48)	3 (21)	4 (9)	1 (109)	2 (7)	3 (3)	4 (1)
Filling	91.73%	81.81%	83.33%	50.00%	88.43%	85.41%	80.95%	44.44%	95.41%	57.14%	100%	25.00%
Direct pulp capping	1.3%	–	–	–	1.65%	–	–	–	0.91%	–	–	–
Pulpectomy	33.91%	12.72%	16.66%	–	14.05%	10.41%	19.04%	–	55.96%	28.57%	–	–
Pulpotomy	13.04%	1.81%	–	–	9.91%	2.08%	–	–	16.51%	–	–	–
Endodontic	13.04%	14.54%	4.16%	–	19.00%	16.6%	–	–	6.42%	–	33.33%	–
Exodontias	38.7%	36.36%	45.83%	60.00%	45.45%	35.41%	42.85%	55.55%	31.19%	42.85%	66.66%	25.00%
DTT + fluoride	86.95%	81.81%	91.66%	90.00%	97.52%	87.5%	95.23%	88.88%	75.23%	42.85%	66.66%	25.00%
Fissure sealant	40.87%	38.18%	58.33%	10.00%	44.63%	41.66%	61.90%	–	36.70%	14.28%	33.33%	25.00%
Scaling and root planing	0.86%	0.00%	–	–	1.65%	–	–	–	–	–	–	–
Stainless steel crown	–	3.63%	–	–	–	2.08%	–	–	–	14.28%	–	–
Space maintenance	–	1.81%	–	–	–	2.08%	–	–	–	–	–	–
MTA apexification	1.30%	5.45%	–	–	1.65%	6.25%	–	–	0.91%	–	–	–

These data come from the reference [16].

**Table 5:** Comparison of prevention program participation between SHCNs and healthy patients.

	SHCNs				Healthy			
	Total	Educ. Health	Maint.	Other	Total	Educ. Health	Maint.	Other
				Treatments				Treatments
**Prevention program**	100%	100%	85.71%	18.4%	100%	97.14%	85.71%	80%
	*n* = 49	**49**	42	9	*n* = 35	**34**	30	28
**No prevention program**	100%	40%	32%	16%	100%	28.12%	21.87%	40.6%
	*n* = 50	**20**	16	**8**	*n* = 32	**9**	7	13
***p*-value**		<0.001 ^f^	<0.001 ^f^	0.755 ^f^		<0.001 ^f^	<0.001 ^f^	<0.001 ^f^

^f^ Proportion test. Educ. Health (Education for health); Maint. (Maintenance).

## Data Availability

The datasets used for the current study are available from the corresponding author upon reasonable request.

## Abbreviations

The following abbreviations are used in this manuscript:

SHCNs	Special Health Care Needs
WHO	World Health Organization
CAMBRA	Caries Management By Risk Assessment
ICDAS	International Caries Detection and Assessment System
